# Heterogeneous constitutional mismatch repair deficiency with MSH6 missense mutation clinically benefits from pembrolizumab and regorafenib combination therapy: a case report and literature review

**DOI:** 10.1186/s13053-021-00165-2

**Published:** 2021-01-09

**Authors:** Tong Xie, Qin Feng, Zhongwu Li, Ming Lu, Jian Li, Analyn Lizaso, Jianxing Xiang, Lu Zhang, Lin Shen, Zhi Peng

**Affiliations:** 1 Department of Gastrointestinal Oncology, Key Laboratory of Carcinogenesis and Translational Research, Peking University Cancer Hospital & Institute, 52 Fucheng Road, Beijing, 100142 China; 2 Department of Pathology, Key Laboratory of Carcinogenesis and Translational Research, Peking University Cancer Hospital & Institute, Beijing, 100142 China; 3 Burning Rock Biotech, Guangzhou, 510300 China

**Keywords:** CMMRD, MSH6, Heterogeneity, ICI, Case report

## Abstract

**Background:**

Germline DNA mismatch repair (MMR) gene aberrations are associated with colorectal cancer (CRC) predisposition and high tumor mutation burden (TMB-H), with increased likelihood of favorable response to immune checkpoint inhibitors (ICIs).

**Case presentation:**

We present a 32-year old male patient diagnosed with constitutional MMR deficiency (CMMRD) CRC whose MMR immunohistochemistry (IHC) revealed inconsistent results from two tumor blocks. Targeted sequencing of two tumor specimens used in MMR-IHC and plasma-derived circulating tumor DNA consistently revealed the detection of bi-allelic germline MSH6 c.3226C > T (p.R1076C) mutation, TMB-H as well as the genetic heterogeneity of the tumor samples. Unexpectedly, both blocks were microsatellite stable (MSS) after PCR confirmation. Interestingly, the patient failed to show response to ICI monotherapy or dual therapy, but clinically benefitted from combined therapy of ICI pembrolizumab plus multi-kinase inhibitor regorafenib.

**Conclusion:**

Our case reported a CMMRD patient with heterogeneous MMR results who showed complicated response to ICIs, highlighting the importance of accurate diagnosis using targeted sequencing with multiple specimens to reveal the possible mechanism of response to ICI in patients with CMMRD.

**Supplementary Information:**

The online version contains supplementary material available at 10.1186/s13053-021-00165-2.

## Background

The DNA mismatch repair (MMR) pathway functions to recognize and repair base pair mismatches that arise during DNA replication and recombination and plays a key role in maintaining genomic stability. Defects in mismatch repair (dMMR) account for approximately 15% of colorectal cancer (CRC) [1]. Approximately 3% of CRC are associated with Lynch syndrome, also referred to as hereditary non-polyposis colorectal cancer (HNPCC), an autosomal dominant disease and the most common inherited form of CRC arising from monoallelic germline mutation in *MLH1*, *MSH2*, *MSH6*, or *PMS2* [2]. Another hereditary syndrome associated with dMMR is constitutional mismatch repair deficiency (CMMRD) syndrome, which is a rare autosomal recessive cancer-predisposing syndrome resulting from bi-allelic germline mutations in MMR genes [3].

National Comprehensive Cancer Network (NCCN) guideline now recommends all patients diagnosed with CRC to undergo MMR and microsatellite instability (MSI) testing to screen for inherited CRC, which is instrumental in identifying Lynch and CMMRD syndromes [4]. Proper diagnosis of these inherited cancer syndromes is necessary for optimal clinical management, including the decision to perform surgery and appropriate adjuvant chemotherapy [2, 5, 6]. However, tumor heterogeneity could contribute to inconsistent MMR-IHC or MSI results, which delays the diagnosis and affects the treatment outcomes [7, 8]. Herein, we report a patient with CMMRD and metastatic CRC resulting from bi-allelic germline MSH6 mutation who had heterogeneous MMR-IHC with high tumor mutations burden (TMB-H) but microsatellite stable (MSS) status that led to his complicated response to immune checkpoint inhibitor (ICI) therapy. We have prepared this manuscript in accordance with the CARE guidelines.

## Case presentation

**Fig. 1:**
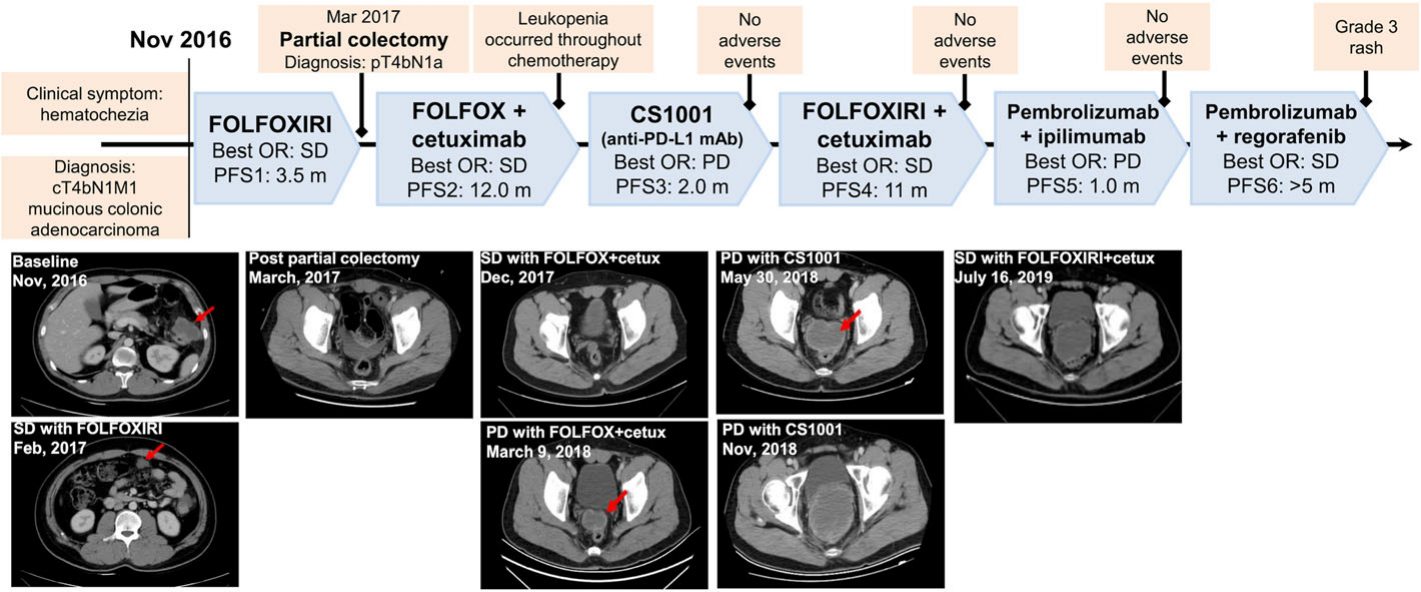
Summary of the treatment received by the patient, including the best objective response (OR) and progression-free survival (PFS) in each line of treatment. Abdominal computed tomography (CT) scans are also provided. Red arrows indicate the primary lesions

**Fig. 2:**
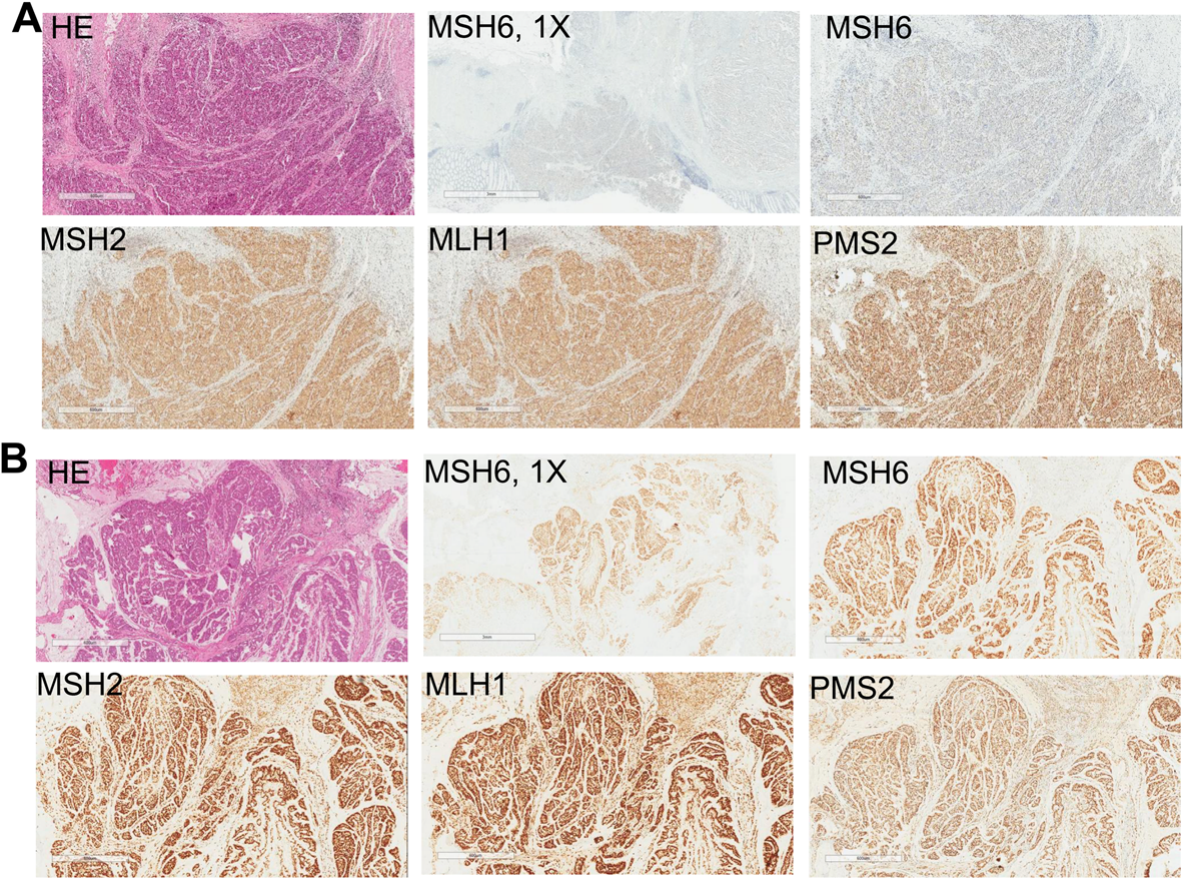
Immunohistochemical staining of MSH6, MSH2, MLH1, and PMS2. One section of the surgical tissue sample was evaluated as (**a**) MMR deficient (dMMR) and the other as (**b**) MMR proficient (pMMR). Hematoxylin Eosin staining, MSH6, MSH2, MLH1 and PMS2 were collected at 4X magnification, unless otherwise indicated (i.e. magnification of 1X for MSH6 in the second panel on top)

In November 2016, a 32-year-old male presented with a chief complaint of hematochezia for 2 years was diagnosed with early-onset colon cancer with peritoneal metastasis (T4bN1M1). Abdominal computed tomography (CT) scans showed a mass in the splenic flexure of the colon with presence of multiple peritoneal nodules (Fig. 1). Additionally, histopathology examination of the tissue biopsy revealed poorly- to moderately-differentiated adenocarcinoma with mucinous features. Palliative first-line FOLFOXIRI regimen (D1: 5-fluorouracil 2.8 g/m^2^ 5g civ46 + oxaliplatin 85 mg/m^2^ 150mg + irinotecan 165 mg/m^2^ 300mg q14d) was administered for a total of 6 cycles achieving stable disease (SD) (Fig. 1). On March 2017, laparotomy and subtotal colectomy was performed with no postoperative complications and confirmed the pathological stage of pT4bN1a. Immunohistochemistry (MMR-IHC) of different sections of formalin-fixed paraffin-embedded (FFPE) blocks of the surgically-removed tumor demonstrated heterogeneity in MMR status, with one section demonstrating complete loss of MSH6 expression and evaluated as MMR deficient (dMMR) (Fig. 2a), while another section demonstrating clonal loss of MSH6 (Fig. 2b, MSH6-1X), but still evaluated as MMR proficient (pMMR) due to moderate staining in some clones (Fig. 2b). However, the MSI status of both blocks was determined as MSS by polymerase chain reaction (PCR)–based analysis (Fig. S1). To further understand the biology of this heterogeneity, two tissue specimens used for MMR-IHC and an additional plasma sample were submitted for comprehensive targeted sequencing with a 520-gene panel (OncoScreen Plus, Burning Rock Biotech). Table S1 summarizes the sequencing results. The tumor mutation burden (TMB) status of all his samples was assessed to be TMB-H. Careful inspection of the mutation profile revealed that the pMMR tissue sample and blood sample had similar mutation profile and different from the dMMR sample. Despite the variability in mutation profile between the pMMR and dMMR tissue samples, a homozygous missense mutation in the exon 5 of *MSH6* gene, c.3226C > T (p.R1076C), was consistently detected in both tissue samples and blood sample. In addition to the consistent wild-type status for *HRAS*, *KRAS*, and *NRAS*, a *SMAD4* R361H was also consistently detected in all his samples. Homozygous germline mutation status of *MSH6* R1076C was also confirmed using lymphocyte-derived genomic DNA analysis (Fig. S2A). Further investigations of his family history revealed that his paternal grandfather and maternal grandmother had stomach cancer and endometrial cancer, respectively (Fig. S2B, I.1 and I.2). He was born from fourth-degree consanguineous parents. His back and abdomen are scattered with multiple café-au-lait macula and neurofibromas with unknown age of occurrence. His daughters were born with blue birthmark. Moreover, genetic testing of his immediate family members revealed heterozygous *MSH6* R1076C, strongly indicating an autosomal recessive inheritance pattern (Fig. S2B). Based on his clinical and genetic data, he was additionally diagnosed with late-onset CMMRD.

Since post-colectomy, he was administered with FOLFOX and cetuximab combination therapy (D1: 5-fluorouracil 2400 mg/m^2^ + oxaliplatin 85 mg/m^2^ + cetuximab 500 mg/kg q14d) for 8 cycles achieving SD. He experienced leukopenia multiple times throughout the treatment process. In March 2018, disease recurrence was suspected after a review of abdominal CT revealed a ring-shaped 50x31mm mass in the peritoneal folds of the anterior pelvic cavity. After reviewing literatures from MEDLINE, we found promising evidence of clinical response to ICI treatment in patients with CMMRD as summarized in Table S2 [9–13]. He was enrolled in a clinical trial for anti-PD-L1 monoclonal antibody CS1001–101 administered at 1200 mg in four-cycle regimen (NCT03744403). The treatment was well tolerated. Despite an unresponsive disease, he chose to remain on the treatment for another 4 cycles until confirmation of disease progression as per Response Evaluation Criteria in Solid Tumors (RECIST) v.1.1. After immunotherapy was terminated, he was administered with FOLFOXIRI + cetuximab combination therapy (D1: 5-fluorouracil 2400 mg/m^2^ + oxaliplatin 85 mg/m^2^ + irinotecan 120 mg/m^2^ + cetuximab 500 mg/kg q14d) from November 2018 to October 2019 until progression. After the failure of one cycle ipilimumab (D1: 50 mg) and pembrolizumab (D1: 200 mg), pembrolizumab plus regorafenib (D1: pembrolizumab 200 mg, D1-D21 regorafenib 80 mg q21d) was administered in the next cycle (November 2019) considering the CMMRD and MSS status of the tumor. After 1 week of combination therapy, regorafenib was suspended due to grade 3 rash. Surprisingly, his clinical symptoms of CRC including abdominal pain were markedly relieved, which led to a remarkable improvement in his ECOG PS from 3 to 0. Serum tumor marker CEA also decreased from 13.18 ng/ml to 4.04 ng/ml within 1 month of combination therapy. After the rash resolved, he resumed regorafenib at a reduced dose of 40 mg qd without any chief complaint. His disease remains stable for 6 months as of the last follow-up on May 25, 2020.

## Discussion

Due to the rarity of CMMRD, early diagnosis is challenging particularly in patients with no family history of cancer and delayed onset of malignancies. In our case, CMMRD was incidentally diagnosed due to the detection of bi-allelic germline *MSH6* R1076C mutation by targeted sequencing. Targeted sequencing allowed the detection of the homozygous mutation which then led to further discovery of the presence of dermatological manifestations (i.e. café-au-lait spots and neurofibromas), family history of cancer, mutation carrier status of immediate family members, and consanguinity of the parents that all together helped in forming a conclusive diagnosis. His family has been appropriately counseled regarding their increased risk of developing cancer. Interestingly, our patient with CMMRD had TMB-H but failed to respond to anti-PD-L1 monotherapy with CS1001–101 and combined therapy of anti-PD-1 and anti-CTLA-4 monoclonal antibodies, which may be due to tumor heterogeneity and MSS status. However, the addition of an anti-VEGFR2 multi-kinase inhibitor regorafenib to the anti-PD-1 therapy was clinically effective for our patient.

CMMRD is associated with poor prognosis and typically manifested as childhood cancers including brain tumors, hematological malignancies, and gastrointestinal tumors [3, 5]. Missense R1076C mutation of *MSH6* gene is located on the ATPase domain and has been evaluated as likely pathogenic in ClinVar database (Variation ID: 89357) with multiple evidence associating this mutation with HNPCC and hereditary cancer-predisposing syndrome. Bi-allelic germline *MSH6* R1076C mutation was also reported in a 45-year-old patient diagnosed with CRC [14]. The phenotype presentation in the patients with mono-allelic or bi-allelic *MSH6* R1076C seemed to be milder compared with those with bi-allelic mutations in other MMR genes [14–16]. Delayed age of onset has generally been observed for *MSH6* mutation carriers as compared to *MSH2* or *MLH1* mutants [17–19]. Moreover, *MSH6* missense mutation carriers were more likely to develop CRC than those with truncating mutations [18]. Consistently, bi-allelic germline *MSH6* R1076C mutation of our patient led to the development of CRC in the third decade of life but without any hematological malignancy or brain tumor. According to the report by Plaschke and colleagues, the hypomorphic nature of *MSH6* missense mutation might only bring about partial abolishment of its protein function [15]. This hypomorphic mutation might also explain the intratumor genetic heterogeneity demonstrated by heterogeneous MMR-IHC status and distinct mutation profile of the two tumor block samples from our patient.

Conventionally, the complete absence of MSH6 nuclear staining in both tumor and normal cells of the colonic mucosa is typically observed in patients with bi-allelic *MSH6* mutation [14–16]. However, MSI is not always present among *MSH6* mutation carriers [2, 18, 20]. Hence, MSI testing in patients with CMMRD, particularly those with MSH6 deficiency may not provide conclusive results. Instead of MSI testing, MMR-IHC could provide more information on the MMR status of patients with CMMRD [20]. These previous reports are consistent with our patient who had MSS and TMB-H, but had heterogeneous MMR-IHC status to which the molecular mechanism is difficult to explain. However, concurrent MSS and TMB-H have been observed in 3% of CRC patients, with approximately 7.3% patients with MSS/TMB-H CRC also harboring *MSH6* mutations [21].

Due to the increase in mutations resulting from the impaired DNA repair mechanisms in CMMRD, ICIs have been explored as a potential strategy in treating cancers in patients with CMMRD [9–13]. In contrast with previously reported data [9–13], no significant clinical response was observed from the primary colonic lesions of our patient. His lack of initial response to ICI may be due to tumor heterogeneity, wherein only the dMMR part of his tumor responded to the ICI while the pMMR region remained immunosuppressed. Heterogeneous MLH1 expression was implicated as the mechanism of failed response to ICI in a patient with dMMR gastric cancer [22]. Interestingly, the addition of regorafenib potentially contributed to making the tumor more “immune reactive” as compared to the use of only ICIs. Despite the MSS status of our patient, his clinical benefit to anti-PD-1 monoclonal antibody pembrolizumab in combination with a multi-kinase inhibitor regorafenib was consistent with ICI response of MSS/TMB-H CRC described in a previous study [21]. Unfortunately, due to financial issues, expression levels of various markers of tumor microenvironment were not analyzed.

In conclusion, our case highlights the importance of the inclusion of targeted sequencing in addition to MMR/MSI testing of patients with CRC to understand the genetic landscape and identify potential hereditary factors. Moreover, the evaluation of multiple specimens is also necessary to generate conclusive results.

## Supplementary Information

**Additional file 1: Figure S1.** Microsatellite assessment by polymerase chain reaction (MSI-PCR of the two tumor blocks used for MMR-IHC were consistently assessed as microsatellite stable (MSS) (A-B). MSI-PCR of six mononucleotide microsatellite markers including NR21, Bat26, Bat25, NR27, NR24, and Mono27, two pentanucleotide microsatellite markers PentaC and PentaD, and an internal control, AmeI. Microsatellite instability with MSI-PCR is assessed as low (MSI-L) if having 1 marker exhibiting changes in length, and assessed as high (MSI-H) when having 2 or more markers exhibiting increase or decrease in length of microsatellite markers. MSS is assessed when all markers have no change. **Figure S2.** Detection of germline homozygous MSH6 missense mutation in the patient. A. Illustration of the homozygous germline *MSH6* c.3226C>T (p.R1076C) of the patient using the Integrated Genome Viewer. B. Pedigree analysis illustrating the family history of cancer and the detection of homozygous *MSH6* R1076C mutation in the patient (III.1) and heterozygous *MSH6* R1076C mutation in the parents of the patient (II.1 and II.2), his wife (III.2) and his two daughters (IV.1 an IV.2). (PPTX 335 kb)

**Additional file 2: Table S1.** Summary of targeted sequencing results from 2 tissue samples and blood sample of the patient. **Table S2**. Review of literature on CMMRD patients treated with immune checkpoint inhibitors.

## Data Availability

The datasets generated during and/or analyzed during the current study are not publicly available, but are available from the corresponding author on reasonable request.

## Abbreviations

CMMRD: Constitutional mismatch repair deficiency
CRC: Colorectal cancer
dMMR: Deficiency in mismatch repair
FFPE: Formalin-fixed paraffin-embedded
HNPCC: Hereditary non-polyposis colorectal cancer
ICI: Immune checkpoint inhibitor
IHC: Immunohistochemistry
MMR: Mismatch repair
MSI: Microsatellite instability
MSS: Microsatellite stable
NCCN: National Comprehensive Cancer Network
PCR: Polymerase chain reaction
pMMR: Proficient in mismatch repair
TMB: Tumor mutation burden
TMB-H: High TMB
