# Supplementary material for: Heterogeneous constitutional mismatch repair deficiency with MSH6 missense mutation clinically benefits from pembrolizumab and regorafenib combination therapy: a case report and literature review
Source: Hered Cancer Clin Pract. 2021 Jan 9;19:7. doi: 10.1186/s13053-021-00165-2 (PMC7797131; doi:10.1186/s13053-021-00165-2)
Supplement: Supplementary file 1 — Additional file 1: Figure S1. Microsatellite assessment by polymerase chain reaction (MSI-PCR of the two tumor blocks used for MMR-IHC were consistently assessed as microsatellite stable (MSS) (A-B). MSI-PCR of six mononucleotide microsatellite markers including NR21, Bat26, Bat25, NR27, NR24, and Mono27, two pentanucleotide microsatellite markers PentaC and PentaD, and an internal control, AmeI. Microsatellite instability with MSI-PCR is assessed as low (MSI-L) if having 1 marker exhibiting changes in length, and assessed as high (MSI-H) when having 2 or more markers exhibiting increase or decrease in length of microsatellite markers. MSS is assessed when all markers have no change. Figure S2. Detection of germline homozygous MSH6 missense mutation in the patient. A. Illustration of the homozygous germline MSH6 c.3226C>T (p.R1076C) of the patient using the Integrated Genome Viewer. B. Pedigree analysis illustrating the family history of cancer and the detection of homozygous MSH6 R1076C mutation in the patient (III.1) and heterozygous MSH6 R1076C mutation in the parents of the patient (II.1 and II.2), his wife (III.2) and his two daughters (IV.1 an IV.2). (PPTX 335 kb) [file 13053_2021_165_MOESM1_ESM.pptx]

## Slide 1
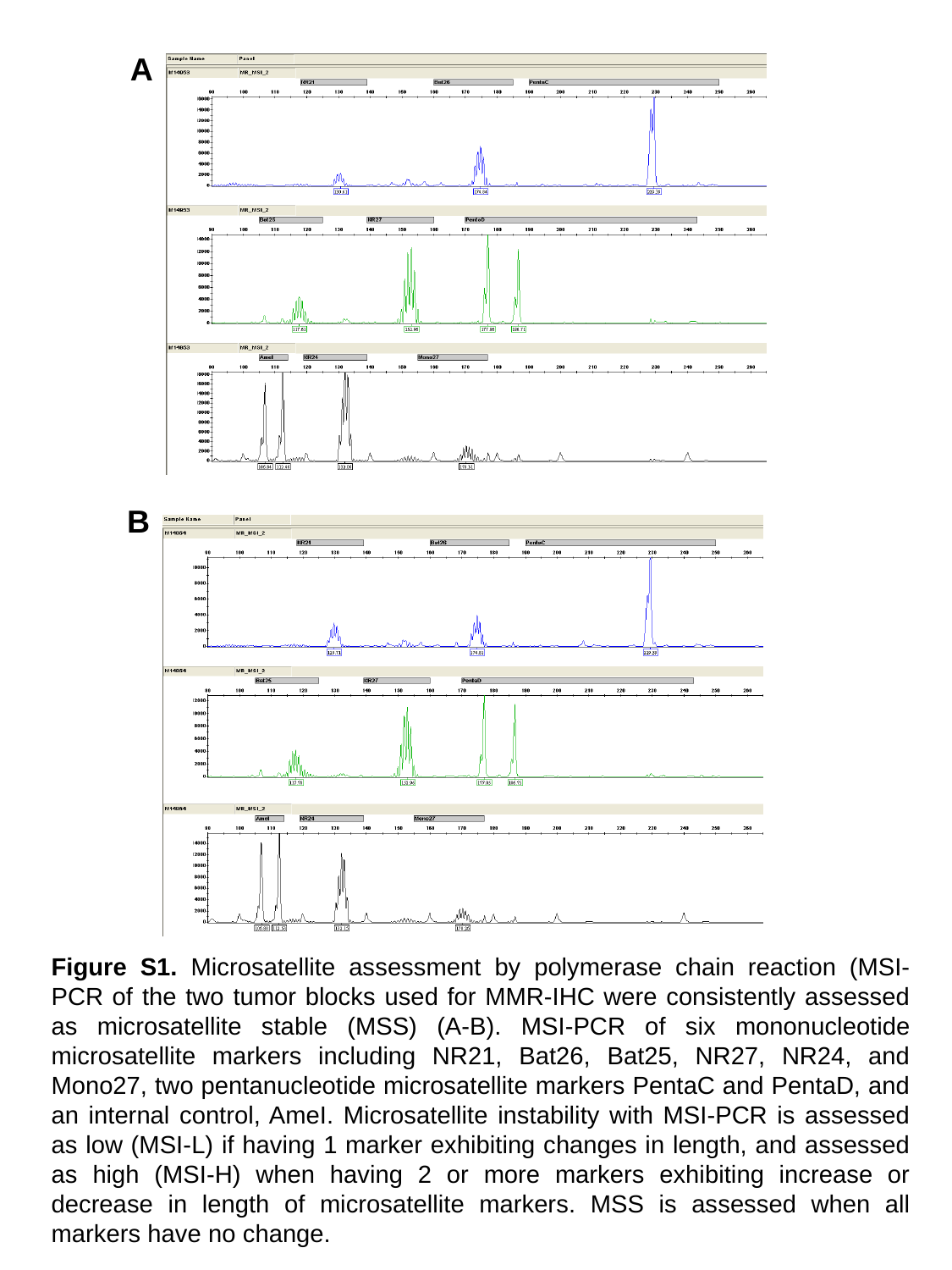

A
B
Figure S1. Microsatellite assessment by polymerase chain reaction (MSI-PCR of the two tumor blocks used for MMR-IHC were consistently assessed as microsatellite stable (MSS) (A-B). MSI-PCR of six mononucleotide microsatellite markers including NR21, Bat26, Bat25, NR27, NR24, and Mono27, two pentanucleotide microsatellite markers PentaC and PentaD, and an internal control, AmeI. Microsatellite instability with MSI-PCR is assessed as low (MSI-L) if having 1 marker exhibiting changes in length, and assessed as high (MSI-H) when having 2 or more markers exhibiting increase or decrease in length of microsatellite markers. MSS is assessed when all markers have no change.

## Slide 2
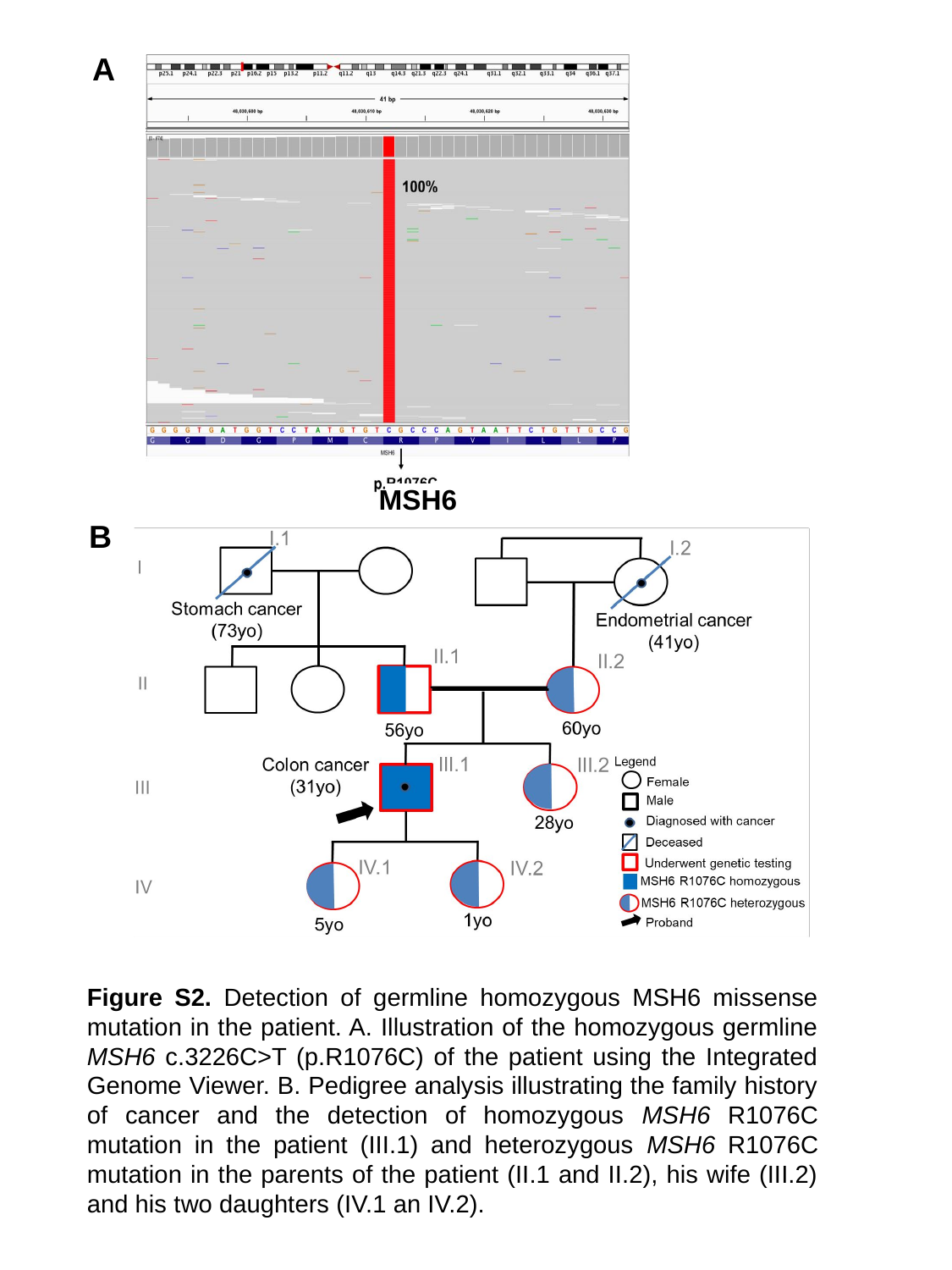

A
MSH6
B
Figure S2. Detection of germline homozygous MSH6 missense mutation in the patient. A. Illustration of the homozygous germline MSH6 c.3226C>T (p.R1076C) of the patient using the Integrated Genome Viewer. B. Pedigree analysis illustrating the family history of cancer and the detection of homozygous MSH6 R1076C mutation in the patient (III.1) and heterozygous MSH6 R1076C mutation in the parents of the patient (II.1 and II.2), his wife (III.2) and his two daughters (IV.1 an IV.2).
