# Supplementary material for: Heterogeneous constitutional mismatch repair deficiency with MSH6 missense mutation clinically benefits from pembrolizumab and regorafenib combination therapy: a case report and literature review
Source: Hered Cancer Clin Pract. 2021 Jan 9;19:7. doi: 10.1186/s13053-021-00165-2 (PMC7797131; doi:10.1186/s13053-021-00165-2)
Supplement: Supplementary file 2 — Additional file 2: Table S1. Summary of targeted sequencing results from 2 tissue samples and blood sample of the patient. Table S2. Review of literature on CMMRD patients treated with immune checkpoint inhibitors. [file 13053_2021_165_MOESM2_ESM.docx]

**Table S1.** Summary of targeted sequencing results from 2 tissue samples and blood sample of the patient

| MMR, MSI, TMB status/ Genomic alterations | FFPE tumor-1 | FFPE tumor-2 | Blood ctDNA |
| --- | --- | --- | --- |
| MMR status based on IHC of same tissue specimen | pMMR | dMMR | NA |
| MSI status based on PCR | MSS | MSS |  |
| TMB (mutations/megabase) | 723.8 | 673 | 242 |
| **NM_000179.2(MSH6):c.3226C>T(p.Arg1076Cys)** | **homozygous** | **homozygous** | **99.93%** |
| **HRAS, KRAS, NRAS** | **WT** | **WT** | **WT** |
| NM_000059.3(BRCA2):c.6952C>T(p.Arg2318*) | 30.66% | - | 1.78% |
| NM_000059.3(BRCA2):c.9218A>G(p.Asp3073Gly) | 29.43% | - | 2.75% |
| NM_000038.5(APC):c.904C>T(p.Arg302*) | 26.91% | - | 2.71% |
| NM_000038.5(APC):c.4348C>T(p.Arg1450*) | 26.00% | - | 2.75% |
| NM_000051.3(ATM):c.8545C>T (p.Arg2849* ) | - | 19.38% | - |
| NM_001184.3(ATR):c.7639C>T(p.Arg2547*) | 30.45% | - | 3.10% |
| NM_005188.3(CBL):c.1837G>T(p.Glu613*) | 31.13% | - | 2.09% |
| NM_005896.3(IDH1):c.395G>A (p.Arg132His) | - | 13.19% | - |
| NM_005896.3(IDH1):c.394C>T (p.Arg132Cys) | - | 9.14% | - |
| NM_018557.2(LRP1B):c.8515C>T(p.Gln2839*) | 23.54% | - | 2.40% |
| NM_018557.2(LRP1B):c.7306G>T(p.Gly2436*) | 46.23% | - | 3.06% |
| NM_018557.2(LRP1B):c.2940C>A(p.Cys980*) | 30.63% | - | 2.98% |
| NM_000267.3(NF1):c.2446C>T (p.Arg816*) | - | 17.37% | - |
| **NM_005359.5(SMAD4):c.1082G>A(p.Arg361His)** | **31.95%** | **39.26%** | **2.98%** |
| NM_005359.5(SMAD4):c.1490G>A(p.Arg497His) | 30.34% | - | 2.56% |
| NM_000249.3(MLH1):c.676C>T(p.Arg226*) | - | 18.85% | - |
| NM_000251.2(MSH2):c.470G>A(p.Gly157Asp) | 27.83% | - | detected |
| NM_000251.2(MSH2):c.1963G>A(p.Val655Ile) | - | 20.85% | - |
| NM_000179.2(MSH6):c.709G>T (p.Gly237*) | - | - | - |
| NM_000179.2(MSH6):c.1082G>A(p.Arg361His) | - | 7.80% | - |
| NM_000179.2(MSH6):c.3223T>C(p.Cys1075Arg) | - | 7.06% | - |
| NM_000179.2(MSH6):c.3460G>A(p.Ala1154Thr) | - | 21.93% | - |
| NM_000179.2(MSH6):c.3505C>T(p.Pro1169Ser) | 28.82% | - | detected |
| NM_000179.2(MSH6):c.3616G>T(p.Ala1206Ser) | - | 19.13% |  |
| NM_000535.6(PMS2):c.1360C>A(p.Leu454Met) | 29.82% | - | detected |
| NM_006231.3(POLE):c.1316T>C(p.Leu439Pro) | 30.64% | - | - |
| NM_006231.3(POLE):c.1394C>T(p.Ala465Val) | - | 15.86% | - |
| NM_006231.3(POLE):c.2147C>T(p.Ala716Val) | - | 11.10% | - |
| NM_006231.3(POLE):c.3109C>T(p.Arg1037Cys) | 11.10% | 6.27% | - |
| NM_006231.3(POLE):c.4988A>G(p.Asp1663Gly) | 31.12% | - | - |
| NM_006231.3(POLE):c.5635C>T(p.Arg1879Cys) | 27.84% | - | - |
| NM_006231.3(POLE):c.6005C>T(p.Ala2002Val) | 31.60% | - | - |
| NM_006231.3(POLE):c.6119C>T(p.Ala2040Val) | - | 6.13% | - |

Note: values in **bold** face denotes detection in all samples; NA, not applicable; gene was not included in the panel used for sequencing. -, not detected. Detected, mutation detected but allele fraction not indicated in the report. Abbreviations: ctDNA, circulating tumor DNA; FFPE, formalin-fixed paraffin embedded; MMR, mismatch repair; dMMR, MMR deficient; pMMR, MMR proficient; MSI, microsatellite instability; MSI-H, MSI-high; MSS, microsatellite stable; TMB, tumor mutation burden; WT, wild-type

**Table S2**. Review of literature on CMMRD patients treated with immune checkpoint inhibitors

| Published literature | Gender | Age | Cancer type | Treatment | Mutation | Response |
| --- | --- | --- | --- | --- | --- | --- |
| Bouffet (2016)^9^ | Female | 6 | Glioblastoma | Nivolumab | *PMS2* c.2117delA | Partial response |
| Bouffet (2016)^9^ | Male | 3.5 | Glioblastoma | Nivolumab | *PMS2* c.2117delA | Partial response |
| AlHarbi (2018)^11^ | Female | 5 | Glioblastoma | Nivolumab | *MSH6* c.1883G>A | Partial response |
| Larouche (2018)^12^ | Male | 7 | Glioblastoma | Nivolumab+ ipilimumab | Not available | Complete response |
| Pavelka (2019)^13^ | Male | 14 | Glioblastoma  Colon cancer | Nivolumab + autologous dendritic cell + radiotherapy with temozolomide | *PMS2* c.2T>A;  *PMS2* c.2521delT | Complete response |
